# Service robotics: do you know your new companion? Framing an interdisciplinary technology assessment

**DOI:** 10.1007/s10202-011-0098-6

**Published:** 2011-11-03

**Authors:** Michael Decker, Rüdiger Dillmann, Thomas Dreier, Martin Fischer, Mathias Gutmann, Ingrid Ott, Indra Spiecker genannt Döhmann

**Affiliations:** Karlsruhe Institute of Technology, Karlsruhe, Germany

## Abstract

Service-Robotic—mainly defined as “non-industrial robotics”—is identified as the next economical success story to be expected after robots have been ubiquitously implemented into industrial production lines. Under the heading of service-robotic, we found a widespread area of applications reaching from robotics in agriculture and in the public transportation system to service robots applied in private homes. We propose for our interdisciplinary perspective of technology assessment to take the human user/worker as common focus. In some cases, the user/worker is the effective subject acting by means of and in cooperation with a service robot; in other cases, the user/worker might become a pure object of the respective robotic system, for example, as a patient in a hospital. In this paper, we present a comprehensive interdisciplinary framework, which allows us to scrutinize some of the most relevant applications of service robotics; we propose to combine technical, economical, legal, philosophical/ethical, and psychological perspectives in order to design a thorough and comprehensive expert-based technology assessment. This allows us to understand the potentials as well as the limits and even the threats connected with the ongoing and the planned implementation of service robots into human lifeworld—particularly of those technical systems displaying increasing grades of autonomy.

## Background

Industrial robots are established in nearly all areas of the manufacturing industry. The automotive industry, just like metalworking, plastics, rubber, timber, and furniture industry, is barely conceivable without industrial robots.^1^ Over the last few years, the world market for industrial robots has grown continuously, however, not in all regions of the world to the same extent (World Robotics 2008). Significant features of industrial robotics include high speed, high torques and forces but also dexterity and precision, enormous power and an almost unlimited repeatability of movements in combination with little downtime, higher product quality, and decreasing equipment costs. From the economic point of view, human output has been replaced by technological output or to put it more simply: Labour costs have been replaced by costs of technology acquisition and operation. Thus, the productivity per worker has been increased continuously. The fact that the complete production process had to be redesigned for the application of robots is not a technical problem at all. A production hall is a confined space, and its interior is optimized for the production process and designed according to the regulations for safe production and occupational safety.

“Service robots” are predicted to have an innovation and market potential similar to the huge impact of industrial robots. First of all, it should be noticed here that the term service robots seem to cover all “non-production robots” (see “first observations on the definition” below). A closer look at the areas of application of today’s service robot systems reveals that out of the 77,000 service robots for commercial applications sold worldwide until the end of 2010, the highest percentage of them is used in the field of defence, rescue, and security (30%), followed by agriculture (25%), especially milking and harvesting robots (World Robotics 2010). These are areas where service robots are successfully operated and supervised by human experts and/or in a dedicated and protected surrounding. Such a robot space can therefore be interpreted as a transition zone between industrial robotics and general service robotics. The robot itself is no longer active within its “safety cage,” which is normally set up for a safe production process. However, outside its cage it is only used in areas where it generally does not come into contact with a third party or does not carry out services around human beings. The person who cooperates with the robot can be trained for this cooperation which turns him—to a certain degree—into a robotics expert himself.

Most services, however, are characterized by the fact that they have to be performed in an environment populated by people (one example might be the cleaning of train stations) or directly involve a human being (museum guide, nursing, or elderly care). The people in contact with these robots can only be trained to a limited extent as robotics experts. Thus, these services implicate that a layperson in robotics can and has to interact with robots and that third parties will encounter a robot’s direct environment. Furthermore, these services are performed in everyday life, which can only be adapted to a limited extent to the application of robots. This combination entails grand challenges, both for the technical realization of service robots and the societal environment where they are employed.

The following first thoughts on a technology assessment of service robots start with initial observations of the subject. How are (service) robots defined in different contexts and which conclusions can be drawn for the technology assessment of service robots? On the basis of case studies, the next section describes different service areas where robots are already in use or where first prototypes are being developed in research laboratories. Finally, those questions that should be considered in an interdisciplinary technology assessment are observed from different scientific-disciplinary perspectives.

## First observations on the definition of service robots

Normally, technology assessment starts with a definition of the subject, here service robotics. Therefore, it is reasonable to get a first overview which definitions exist and in which context to ensure their compatibility or, if necessary, to be able to justify a plausible distinction. As mentioned already, service robots are defined as “non-industrial robots.” The International Federation of Robotics (IFR) states on its website:

Service robots have no strict internationally accepted definition, which, among other things, delimits them from other types of equipment, in particular the manipulating industrial robot. IFR, however, has adopted a preliminary definition:

A service robot is a robot which operates semi- or fully autonomously to perform services useful to the well-being of humans and equipment, excluding manufacturing operations.^2^

We will first focus our attention on robots in general. A closer look at the history and development of robots, described—among others—by D. Ichbiah, reveals that robots set a milestone in the progressive human attempt to create machines that support and enable people to perform better, take over some of their workload, cooperate and interact with them, and finally are subservient and undemanding servants.^3^ The term robot harkens back to the Czech author Karel Capek. In his native language “robota” means servant or obedient worker. This would mean that the service aspect is already included in the term robot. A technical definition can be found in the VDI guideline 2860 (Assembly and handling units; handling functions, handling units; terminology, definitions, symbols):

A robot is a freely and repeatedly programmable, multifunctional manipulator with at least three independent axes to move material, parts, tools, or special instruments on programmed, variable tracks to fulfil various tasks.^4^

The following definition adds the fact that the robot is “supporting the human being” to the technical description:

Robots are sensorimotor machines to extent the human ability to act. They consist of mechatronic components, sensors, and computer-based control functions. Robots are extremely complex; more degrees of freedom as well as the variety and extent of their forms of behaviour and body distinguish them considerably from other machines.^5^

This definition attempts to distinguish robots from simple finite state automats by pointing out the larger number of degrees of freedom, their multimodal man-machine interface, and the variety and extent of their forms of behaviour. An automated garage door or a bread maker features mechatronic components, sensors, and a control function, but they would not be complex enough according to the above definition. A modern aircraft or automobile is also equipped with the technical elements mentioned in the definition and is much more complex. Therefore, they could be classified as robots. Something similar is true for Ambient Assisted Living devices (AAL devices) and/or different applications of ubiquitous computing. According to the definition above, they could also be classified as robots (hidden robots), even if they are normally not included in this concept.

Turning to the service robots now, we should first of all define if the term “service” is used according to everyday language usage or in an economic science context.^6^ “Service” in the colloquial sense can be described as “the sum of all human work steps […] that satisfy needs directly without the use of material goods” (Maleri 1997, p. 6). So the focus is on executing a service, accomplishing, or acting in general instead of material goods. From the economic point of view, the term “service” seems to be insufficiently defined. This might be due to the extremely multifaceted types and forms of services and the fact that they cannot be clearly distinguished from contributions to humans or to material goods. According to Maleri, “services” are intangible assets produced for a third-party need using external production factors. At the same time, “production” is defined as the directed fabrication of material goods and services using other material and immaterial goods and is divided into different (economic) sectors:

- primary industry (primary production) that covers agriculture, forestry, fishing, hunt, and sometimes also mining;
- secondary industry (secondary production) with the manufacturing industry and craft, as well as
- the service industry (tertiary production) that includes all other economic sectors like commerce, banks, insurances, restaurants, consulting, and entertainment.

In addition, a further division of the tertiary sector into a quaternary or quinary sector for information and leisure is being discussed (Maleri 1997, p. 10). According to Clark, a distinction can also be made between direct and indirect services. While the end-user is the direct user of direct services, indirect services are production factors. Intangible real goods can be subdivided into performance, services, information, and rights. Especially the distinction between performance and services seems to be relevant in the context of robotics and highlights again the fuzziness of the everyday usage of the term “service.” Performance is understood as the physical and mental human performance provided by households. Although it is also a characteristic of numerous services, in the end, it is an isolated, non-complex offer, that is, not a good resulting from the use or the combination of several production factors (auxiliaries, supplies, current assets, planning, organization,…) (Maleri 1997, p. 23 and p. 53).

The definition of service robots reveals the reference to the economic concept of services. In 1994, the Fraunhofer Institute for Manufacturing Engineering and Automation (Fraunhofer IPA) phrased the following definition of the work of the Institute which is still valid today (Schraft et al. 2004, p. 9):

A service robot is a freely programmable mobile device carrying out services either partially or fully automatically. Services are activities that do not contribute to the direct industrial manufacture of goods, but to the performance of services for humans and institutions.

**Fig. 1:**
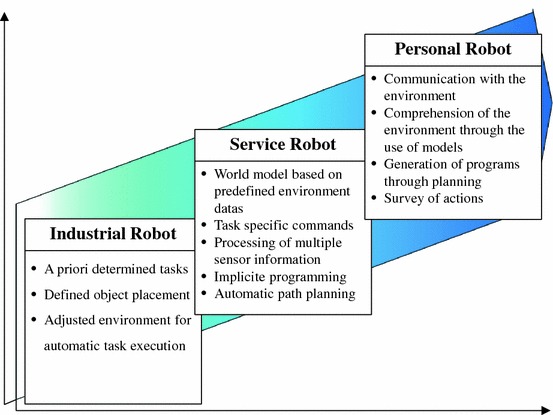
Schraft et al. 1993: differentiation between industrial and service robots regarding the degree of autonomous execution of tasks

The following definition highlights the specific characteristic that distinguishes service robotics from industrial robotics. “Robots in the service sector will differ from industrial robots; they will be individually designed for the execution of a given task taking place in a specific environment.” (Schraft et al. 1993, cf. Fig. 1).

Schraft et al. also mention so-called “personal robots” but do not specify them in detail.

Before we conclude our considerations on the definitions of service robotics with a tabular classification (Table 1), we will quote a definition of Engelhardt and Edwards after Hüttenrauch (2006, p. 3):

**Table 1:** IFR: list of service robots by tasks

*Personal/domestic robots*
Robots for domestic tasks
Robot butler/companion/assistants/humanoids
Vacuuming, floor cleaning
*Lawn mowing*
*Pool cleaning*
Window cleaning
Entertainment robots
Toy/hobby robots
Robot rides
Pool cleaning
Education and training
Handicap assistance
Robotized wheelchairs
Personal rehabilitation
Other assistance functions
Personal transportation (AGV for persons)
Home security & surveillance
*Professional service robots*
Field robotics
Agriculture/*milking robots*
Forestry
Mining systems
Space robots
Professional cleaning
Floor cleaning
Window and wall cleaning (including wall climbing robots)
Tank, tube and pipe cleaning
Hull cleaning (aircraft, vehicles, etc.)
Inspection and maintenance systems
Facilities, plants
Tank, tubes and pipes and sewer
Other inspection and maintenance systems
Construction and demolition
Nuclear demolition & dismantling
Other demolition systems
Construction support and maintenance/construction
Logistic systems
Courier/mail systems
Factory logistics (incl. automated guided vehicles for factories)
Cargo handling, outdoor logistics/other logistics
Medical robotics
*Diagnostic systems*
*Robot assisted surgery or therapy*
Rehabilitation systems
Other medical robots
Defence, rescue & security applications
Demining robots
Fire and bomb fighting robots
Surveillance/security robots
Unmanned aerial vehicles/unmanned ground based vehicles
Underwater systems
Mobile platforms in general use
Robot arms in general use
Public relation robots
Hotel and restaurant robots
Mobile guidance, information robots
Robots in marketing
Others (i.e. library robots)
Special purpose
Refueling robots
Others
Customized robots
Humanoids

[…] systems that function as smart, programmable tools, that can sense, think, and act to benefit or enable humans or extend/enhance human productivity.

The word “think” in this definition explicitly points out cognitive skills and reasoning about the task to be done. This definition of service robots can be combined with definitions of robots in general which refer less than the ones mentioned above to the technical equipment as central defining element. Trevelyan (1999), who refers to “intelligence,” can be quoted as an example: “Robots are intelligent machines capable of extending human skills.” This definition brings up other terms like “intelligence,” “autonomy,” “cooperation,” etc. which have to be discussed in the context of an interdisciplinary reflection on service robotics.

### Case studies

If the definition of service robotics is interpreted strictly as including all applications that do not take place in a production hall, this results in a rather broad range of applications.

One of them could be (1) robotics in agriculture. This includes production facilities that are commonly not referred to as production halls. Of course a cowshed can be adapted for the use of a robot, for example, a milking robot. But nevertheless, the cows as elements of the “production” which cannot be comprehensively described in the technical sense remain a technical challenge. Autonomously driving tractors or harvesting machines are another example for robot systems in agriculture. They are used outside the halls for farm work. Therefore, service robotics in agriculture can be understood as an “extension of industrial robotics.” It is used in a professional environment, that is, in a manufacturing business. The staff can be comprehensively trained for the work with service robots, including an examination for the “operation of a service robot.” The robot is used in a protected private, but also publicly accessible space, like the cowshed or the own field. Therefore, an encounter with uninvolved people (customers at the farm, bicyclists on farm tracks, etc.) cannot be obviated, but it is also impossible to operate the robot within a safety cage due to the type of application. With approx. 15,000 systems sold worldwide until 2008, this is an area where robots are already in use. Therefore, it is also possible to gain empirical insights: Forums like “Indoor agriculture, Buildings and Plants” on the website landlive.de provide discussions on “Milking robots—yes or no?”.

At the conference on “Automation and Robots in Agriculture” of the Association for Technology and Structures in Agriculture (KTBL), Dr. Arno Ruckelshausen (2010) stated that the development of autonomous field robots marks the next step of the inevitable automation of agricultural technology. In the years after the introduction of the first marketable prototypes for specific tasks like weed and pest control and the respective information on the economic, ecological, or social framework conditions, a coexistence of large agricultural machines and small field robots has to be expected.

All this started more than 20 years ago in greenhouses with vision-guided harvesting robots for tomatoes, cucumber, and even for asparagus. Soon afterwards, the robots also found their application field in horticulture as harvesting, robots for citrus fruits and apples. Already in 2006, Baerveldt and Astrand of Halmstad University were able to introduce one of the first weed-killing robots (Grift 2007). To date, other developers also try to establish such weed-killing robots as fully functional autonomous machines on the fields. Projects like BoniRob, an autonomous field robot to collect measured data of individual plants developed by the University of Applied Sciences Osnabrück in cooperation with Bosch and the Amazonen-Werke, have to be mentioned among others. Some of these machines have later been used for targeted weed killing or fertilizing of individual plants, or “weed killer,” a robot developed by the École Nationale d’Ingénieur de Brest and AGRO DEAL for weed killing in row crops can be mentioned here (cf. Fig. 5).^7^^,^^8^

(2) Driver assistance systems are another example for a further step of robots “leaving the factory floors.” The system of individual traffic can be described as a grown infrastructure with various established rules. Every vehicle has got an owner or driver. He has several obligations like reading the manual, ensuring the roadworthiness of the vehicle, regular general inspections, third-party insurance, and driver’s licence, etc. In contrast to agriculture, “everyone” should be able to operate a motor vehicle. This means vice versa that a training as “robotics expert” is only possible to a limited extent. In addition, the vehicles are operated in the public, where “third parties”—pedestrians, bicyclists, etc.—can also be encountered.

The set-up and further development of driver assistance systems, intelligent transport systems, and telematics systems (including, among others, Adaptive Cruise Control (ACC) to contribute to active road safety or Vehicle-to-Infrastructure Systems (V2I) to allow a communication (data transfer) between vehicle and infrastructure to control the traffic) are based on the following reasons:

- Road safety: Technical systems shall improve the active road safety.
- Optimization of traffic flow: Technical systems shall optimize both the economic and ecological aspects of the motorized individual traffic. Road traffic shall become more efficient, and congestions shall be avoided.

The Federal Ministry responsible for transport (then BMVBW) states a need for research concerning road safety (BMVBW 2001, p. 17f., transl. by the authors):

Even more than to date, vehicle technology shall be used to avoid accidents (active safety) and to minimize the consequences of accidents, i.e. to improve the passive safety. The use of telematics systems in road traffic will contribute to the avoidance of accidents.

Taking a closer look at the causes of the accidents, it can be noticed that 15% were attributed to ignoring the right of way, 14.35% to inappropriate speed, and 11.5% to not respecting the safety distance. So 40% of all road accidents with damage to persons result from these three failure causes.^9^ An analysis of rear-end collisions with injured people made by BOSCH revealed that 20% of the drivers applied the brakes too late, 50% did not brake hard enough, and 30% did not brake at all.^10^

Current and future technology could be used to automatically keep the safety distance to the vehicle in front and adapt the speed to the traffic situation and traffic rules, that is, to decelerate the vehicle and thus possibly reduce the number of collisions.

Concerning the traffic flow, it can be stated that congestions are an everyday phenomenon in road traffic. In the German state of North Rhine-Westphalia, there are at least 100 congestions per day.^11^ Congestions often occur at crossroads and traffic lights, in bad weather, because of road works or accidents. According to M. Schreckenberg (2007), every German citizen is caught up in congestion for an average of 2.4 days per year. The cost for a 3-h congestion with a length of 4 km on a two-way “Autobahn” (German freeway) amounts to € 20,000–100,000.^12^

Dietmar Bachmann, member of the State Parliament of Baden-Württemberg, reported during the 32nd session of the Parliament on 11 September 2007 that “the cost for congestions that occur in road traffic due to the waste of fuel […] [amount] to approximately 12 billion € per year. The total economic loss due to congestions on our roads amounts to more than 100 billion € per year.”^13^

While driver assistance systems represent the use of robots “with people” (the driver), self-driving robot systems are also conceivable in public road traffic. Of course the driverless subway and airport transportation system is an exception. But service robots that do the shopping (fetch and carry tasks) also have to move in public places. “RoboCup Search&Rescue” can be given as a “benchmark” for this kind of robot systems where robots—although in disaster operation, but still—have to get along in normal/defective infrastructures. The following research objectives are related to this:^14^

- Collection, accumulation, relay, selection, summarization, and distribution of necessary information.
- Prompt support for planning disaster mitigation, search, and rescue.
- Reliability and robustness of the system during routine and emergency operations.

Given the above listed requirements, the intention of the RoboCup Search&Rescue project is to promote research and development in this socially significant domain at various levels involving mixed multi-agent team work coordination, physical robotic agents for search and rescue, information infrastructures, personal digital assistants, a standard simulator and decision support systems, evaluation benchmarks for rescue strategies, and robotic systems that are all integrated into a comprehensive systems in future. This problem introduces researchers into advanced and interdisciplinary research themes. As AI/robotics research, for example, behavior strategy (e.g. multi-agent planning, realtime/anytime planning, heterogeneity of agents, robust planning, mixed-initiative planning) is a challenging problem. For disaster researchers, RoboCup Search&Rescue works as a standard basis in order to develop practical comprehensive simulators adding necessary disaster modules.^15^

The provision of services by robots in private life (3) is given as third example. It will be described in more detail here since it marks the other end of the above-mentioned spectrum of service robotics and provides good starting points for multidisciplinary discussions. Robotics provides applications for all age groups: Toy robots, entertainment robots, kitchen aids, assistant robots, care robots for disabled, elder and sick people, etc. These robots are applied in private life. Of course it has to be decided for each individual case to what extent a private user can be expected to do special training for the use of a service robot. However, concerning children and sick persons, we have to assume limited or reduced cognitive abilities that make it difficult for them to read the user manual. Therefore, they might not be able to make an efficient use of the system, or to comply with the specified service intervals and maintenance, etc. This field of application puts high demands on the robots. They have to be able to move around safely in an unknown environment (flat) that is not geared to them and perform a number of different tasks. If the programming efforts prior to the initialization and start of robot operation should be still acceptable for laypersons, most of the adaptation to the new environment and the new user has to be done by the robot itself or with the help of internet-based services. This technical problem is even more critical for older users and those in need of care, since they are often cognitively unable to instruct the robot system appropriately or they overestimate the robot skills and capabilities.

Thus, the interactive “intuitive” handling of the robot system is becoming more important, and therefore, at least according to some supporters of humanoid robot systems, they should look as humanlike as possible (Behnke 2008, p. 6). This can become relevant if robots are applied to perform social services, for example, in the field of human care.

Robots for children are a separate field of application in the private sector with a broad variety: Toy, artificial pet, learning aid, babysitter, robot nanny, substitute teacher, etc. The term “edutainment” (Schraft et al. 2004) combines elements from two areas that are intended for completely different purposes: “Education” in the form of training, teaching, and learning is often the exact opposite of “entertainment.” To categorize them as toy robots (Ichbiah 2005) is also difficult since this includes a number of very different robot systems. Therefore, some robot systems will be briefly described as examples.

“Pleo” is a robot in the shape of a dinosaur baby (Camarasaurus) developed by Innvo Laps. Being equipped with two microphones, two loudspeakers, a camera, and approx. 14 sensors at head, chin, shoulders, back, and legs under the skin, Pleo can get in contact with the outside world. What is special about Pleo is that it can interact with its environment and show its virtual “feelings” by certain facial expressions, gestures, or sounds. Pleo “is hungry,” “wants to play,” “needs attention,” and “is tired.” Interaction with Pleo, like stroking his head or back, touching its legs, simulating to feed it, playing with it, talking to, or ignoring it, satisfies its needs and thus, it develops, “learns,” is being “educated.”

A number of robotic building sets were developed in the tradition of LEGO^®^ or fischertechnik design and building sets (ROBO by fischertechnik and MINDSTORMS 2.0 by LEGO^®^).^16^ The focus of these products is on programmable logic components that are equipped with numerous interfaces to sensors and actuators as well as interfaces to a computer. They can be used for the quick and easy assembly of artefacts like pathfinders, sorting systems, and other technical systems. With the appropriate directions and guidance, these building sets will give children from the age of 8 years the opportunity to gain their first experience in the field of robotics. By now, a number of challenges have been established around schools and universities, for example, FIRST^®^ LEGO^®^ League,^17^ where the teams have to complete various tasks with their self-made robots. There are even special activities for girls like ROBERTA^18^ to awaken especially the girls’ interest in (this) technology.

PaPeRo (Partner-type Personal Robot), a robot developed in Japan, can be described as a further development of babyphones—some kind of babysitting robot. “Our continuing research & development is geared toward creating communication robot that can live with us and serve as companion to all of us including children and the elderlies.”^19^ PaPeRo features voice recognition, voice response, facial recognition, face tracking, and touch sensors. It is mobile, recharges its batteries automatically, can imitate sounds, and play a quiz. In addition, this robot can be used to send messages. It can be controlled remotely, and its software can be enhanced by open access. PaPeRo is intended as a companion for children.^20^

Different types of household robots have already been developed, above all those which are already “in use” like vacuum cleaning or lawn-mowing robots. The kitchen seems to be an area where robot assistance is especially welcomed. In contrast to the vacuum cleaning robot that replaces the human being as operator of the vacuum cleaner, here the focus is on the cooperation with humans in everyday scenarios. The humanoid robot ARMAR that was developed by the Collaborative Research Center (SFB) “Humanoid Robots—Learning and Cooperating Multimodal Robots”^21^ is to be applied in the kitchen. The aim of the project is to develop concepts, methods, and concrete mechatronic components for a humanoid robot that shares its working and activity space with humans. In order to be a helpful assistant in everyday life, the robot system must have many complex abilities and characteristics: ARMAR 3 is, for example, able to fetch and carry small items like cups, mugs, a pack of rice, or a juice box. It can also bring a particular drink from the fridge, lay the table, or load and unload the dishwasher. Learning by demonstration is a central element of the cooperation between human and robot in SFB 588.

The scientific competition “Robocup@Home” also puts the application area “at home” into a scientific focus. It is about household service robots; the infield testing takes place in realistic environments (living room, kitchen, or even garden). The robots are completely autonomous and equipped with “intuitive” human–machine interfaces like natural language and gestures. The following topics are of interest for the robotic researchers at Robocup@Home:^22^

- cooperative human–robot interaction,
- cooperative human–robot task solving,
- manipulation of domestic objects such as doors, kitchen utensils, and glasses, etc.,
- navigation in home environments,
- high-level cognition for robots in domestic environments,
- applications for domestic service robots,
- benchmarking domestic service robots,
- long-lasting robotic experiments in domestic environments,
- acceptance of robots in households.

Different service robots for the support of elder or sick people are either in development or already in prototype status. Smart environments or hybrid living spaces including robots as a standard device are proposed. Since these robots have already been described in numerous other publications and are a recurring topic of public discussion, we would like to refer to the relevant literature here.^23^

The three case studies described above are good examples for the “problem field” service robotics since they mention different service contexts. They can be distinguished by the professionalization of the human being who is using the technology. The professionalization (in the sense of being able to be trained or even qualified) decreases from the use in agriculture to household applications. They also differ regarding the environment where the robot is used, that is, in public or in private. Finally, they can be classified according to the economic environment [business-related use (1, 2), private use (2, 3)] and the physical and mental abilities of the users, which might be below the “normal” level in case study 3.

## Multidisciplinary questions

In this section, we will discuss the questions from the respective disciplinary perspective.

### Technological perspective

The successful provision of a service is already a big technological challenge. This can be compared with a “checklist” that can be compiled for a particular service. The service “vacuum cleaning” is provided successfully if the floor is clean, and if this is done without damaging the furniture, without making too much noise, within a reasonable time, etc. If the vacuum cleaning robot has met these requirements, the service is—in technical terms—performed successfully. A basic requirement in the private environment is that the robot has to be able to find its way “autonomously” in a surrounding that has to date been unknown and that it can adapt to the environment in which it has to perform its service. To summarize it briefly: The robot has to be enabled to learn its task and its environment. Here, we take different approaches, which aim, among others, at learning “like human beings” (“learning like a child” (Xpero project),^24^ “learning by demonstrating” (ARMAR project), etc.) where “trial and error and imitation” play a central role. A humanoid stature (torso, head, arms, and legs) is often considered to be an advantage for learning. On the one hand, it animates people to interact with the robot; on the other hand, the robot is “physically” adapted to an environment that is optimized for human beings (steps adjusted to the length of human legs, doorways, signs at “eye level,” etc.) (Behnke 2008, p. 5). While concerning the last aspect “humanoid” just means having human dimensions and movement abilities as well as multimodal communication capabilities, making the robot even more manlike can be an interesting aspect to support learning. Then, we would be speaking of android or gynoid robots with a “confusingly similar” appearance to human beings. This “being like humans” could become relevant when it comes to the technical realization of so-called soft skills like friendliness, helpfulness, which are related to the provision of services. It is also important that the human being on the one hand, who is capable of integrating his knowledge and using his experience, and the specialized, skilled humanoid robot on the other hand, share their information by exchanging and thus updating their respective knowledge.

### Economic perspective

Major trends provide various opportunities for the use of service robots: Since the industrial revolution, the importance of the service sector has steadily increased, and in Germany, for example, its contribution to overall added value generation as well as to employment amounted to almost 75% in 2009.^25^ A similar development might also be observed in other high-technology countries. Structural change from the primary to the secondary and tertiary (the service) sector is accompanied by a transition towards knowledge-based societies. People are well educated, and the citizen’s knowledge and their dynamics are key factors and drivers within innovation processes, especially in application fields where ICT is playing a major role, user-driven innovations are prevalent. As a consequence, in the context of service robotics, individual skills significantly affect both supply-side and demand-side aspects.

The major distinction between service and industry robots is based on the characteristics of services: They are immaterial and thus experience goods whose quality can only be assessed once they are actually used by the customer(s). The simultaneity of production and consumption as well as the consequential direct relation between service provider and customer is the reason why services cannot be stored, exchanged, or sold again. Due to the human interaction during the performance of the service, the possibilities for standardization are rather limited. At the same time, standardization is a major prerequisite for the application of service robots in both individual and professional use.

The introduction of service robots raises several questions, including some topics concerning standardization and patenting. Questions that have to be addressed in order to estimate the potential of service robotics include: What is the incentive for individual actors to develop or use service robots (e.g. lack of nursing staff in an “aging society” and/or the resulting profit opportunities)? Which costs incur throughout the innovation process of the robots (technical and non-technical costs)? Their use requires adjusting them to existing environments, hence aside from the use of “complementary” qualified staff that operates the robot, also adjustment costs, for example, for the modification of the surroundings in which the robots become active, have to be borne. Are those who bear the costs also the ones who receive the revenues? Furthermore, it is important to identify the stakeholders and the relevant markets. The acceptance of technologies and thus their demand may be higher in technophile economies (Japan is generally considered as being one of them) than in more conservative ones. Are there certain countries that are supposed to become lead markets in that field? An overall assessment of the potential, for example, for the labour markets, does not only consider those jobs which might be replaced by robots but also includes especially those which are newly created in the course of innovation. And finally, what are the preconditions of the national or regional innovation systems (including the legal and political framework) where robots are developed?

### Legal perspective

Depending on the field where service robots are used, different legal questions arise. We can distinguish between those concerning the relation citizen–citizen (civil law) and others concerning the relation between the state and the citizen (public law). As a regulatory tool, public law restricts economic activities that collide with the rights and legal interests of others or the common good. Here, one major problem consists of governmental decisions under uncertainty. If and how the legislative authority intervenes depends on prognostic assumptions whose future fulfilment is uncertain. It is not foreseeable if and to what extent and in which social contexts service robots are accepted and used and thus change social systems, social perception, as well as demand changes, for example, in the existing infrastructure, in social welfare, and healthcare provision and finally damage regulation. It is also unclear whether existing requirements for production safety which are already covered by the existing legal foundations of private liability law are applicable and sufficient to cover potential harm to people and objects and whether they set the right incentives: Do we assume a generally dangerous activity—in line with the strict and far-reaching liability regulations, for example, of genetic engineering or atomic energy which calls for an absolute liability? There is also the need to consider secondary objectives of liability: The promotion of any innovation can only be successful if the chosen liability scenario does not regulate the entrepreneurial (and private) development in such a strict way that further developments do not pay off. More importantly, individual legal requirements may interfere with innovative ideas: Social law, for example, which is especially relevant for services in the field of health and care (age, disability, and sickness), demands attention to a number of special requirements, some of them induced by constitutional law. They differ significantly from the legal framework service robots encounter in professional environments, for example, in agriculture.

From the perspective of civil law, where the relation citizen–citizen is in the focus of legal considerations, it is mainly a question of liability of those who plan, manufacture, sell, and finally use service robots to the integrity of legally protected goods of those people who get in contact with service robots. Here, the existing regulation instruments should be made applicable to the new problems of warranty and hazard. This refers to the drafting of contracts, especially regarding the risk allocation in the General Terms and Conditions as well as general questions of liability for damages to third parties. The formulation of due diligence and liability standards is a central element here. If the requirements are too strict, this will impede—or even prevent—the manufacturing, distribution, and use of service robots; if the requirements are too low, the use is seen with even more scepticism the more defect-prone the relevant service robots turn out to be. However, it should be noted that civil liability rules are only one means of reducing the risks associated with the operation of potentially dangerous technology. Ideally, in regulating such technology, civil law rules should be combined with, and complemented by, public law rules, which aim at preventing or, at least, reducing technology risks in the first place. Additional issues are raised if service robots are autonomously adaptive and can react with other robots or the environment in general in a way that is not predictable in detail. This leads to the question to what extent damages caused by the operation of such robots can still be meaningfully attributed to the person(s) operating the robot, or whether new rules of accountability, such as the creation of an independent legal “liability” of these novel mechanical “beings,” are called for. So far, this issue has only been discussed for software agents but not yet for service robots.

### Psychological perspective

The design of the “interface” between human and robot is a central element of service robotics. The case studies include many facets: from “integrated into the overall system automobile,” via “faceless robot systems” (milking robots/autonomously driving tractors or harvesting machines) through to a really manlike humanoid robot system. Within these human–robot systems exists a clear assignment of roles and functions of human and robot which answers the question which tasks are better performed by the robot and which should be done by the human being—from the psychological point of view one of the most important questions in contract design. However, this division of tasks bears the risk that the human being is only taking over those (remaining) tasks which the robot cannot carry out. This question is also relevant in non-working contexts—that is in private life: Which tasks could and should remain with the human, which tasks should be taken over by the robot?

Depending on the general allocation of tasks between human and robot, (ergonomic) issues that can be assigned to the human–machine communication have to be dealt with from the psychological point of view. Concerning the dialogue principles of programs, DIN EN ISO 9241-110 lists “suitability for the task,” “self-descriptiveness,” “controllability,” “conformity with user expectations,” “error tolerance,” “suitability for individualization,” and “suitability for learning”(cf. Schneider 2008). These issues also play important roles in service robotics, where decisions have to be taken that affect the handling and user-friendliness of the robot system.

When it comes to making technical systems user friendly, the criterion of “intuitive” handling is of great relevance today, for example, in the context of mobile phones. In the field of service robotics, this issue gains a special relevance: The aspect of “intuitive” handling focuses on the “appearance” of the robot, which brings humanoid robot systems into play. People tend to personalize things and thus also technology. So the question is also how humanoid should a robot system be for a special task, which is, like in our example, a service task and being performed in peoples’ privacy. The hypothesis of “uncanny valley” (MacDorman and Ishiguro 2006) suggests that an appearance that supports cooperation can turn into an “eerie” perception, which is counterproductive for user-friendliness.

The industrial psychological consideration suggested here puts special emphasis on the allocation of tasks between human and robot in the cognitive field. Basically, this is a question of sharing responsibility and interaction between human and artificial intelligent systems: When may and should the robot provide a service autonomously and proactively based on the assessment of a situation without having received specific instructions to become active? When is it allowed to correct assumed mistakes in the action of humans without explicit order? Is a humanoid robot capable of interacting with its environment in a social manner? This is a psychological issue since questions concerning the ability to judge and mental capability play a role here; however, it also touches the ethical and legal dimensions of technology assessment.

### Philosophical and ethical perspectives

From an ethical point of view, the focus is on the desirability of certain technical solutions regarding their *reasonability*. These questions will be discussed hereafter on the example of robots in caregiving/medical services.

Today, services in the field of caregiving, or medical care in general, are typically provided by human beings. However, the statistics for industrialized countries predict a demographic change, which means that the number of people in need of care will be growing in the foreseeable future, while the number of caregivers is going to decrease. Against this background, it could be desirable for a society to develop service robots for care (Sparrow and Sparrow 2006). Their use can be planned to different extents, with the spectrum reaching from simple assistance in caregiving to “real” care robotics in the narrower sense.

Ethical questions on the desirability, which are connected to such scenarios, usually refer to the classical questions of ethics of technology. This is about the scientific reflection of moral statements that are often cited as arguments for the acceptance or the rejection of the use of technology. Cost–benefit considerations also play a role here. The questions are then answered with reference to procedural utilitarian, discursive, or participatory approaches. Such ethical considerations in the narrow sense form the standard repertoire of ELSI concepts which are also common for robotics and autonomous systems in use in parallel to ongoing research (cf., e.g. Royal Academy 2009). A comprehensive ethical reflection also includes methodological questions aiming at the determination of what should be considered succeeding or even successful support, replacement, or surpassing of human performances, abilities, or skills. Then, the design criteria for the adequacy of the description of robotic systems that replace human actors gain centre stage (cf. Gutmann 2010; Sturma 2003 on this). The methodological reflection focuses on an equalization of human and machine including a thorough analysis of the limits of technical systems engaging into decision-making, which would address them as potential moral agents (s.e. Asaro 2006). This is followed by the differentiation of human–machine, machine–human, machine–machine, and human–human interaction where a differentiation of connection, interaction, and interface could become relevant, terms that are often used synonymously (cf. Hubig 2008). Only such a clarification can provide information on the logical grammar of the “as-if” structure and thus the attribution of emotive, volitional, and cognitive terms to robotic systems. A systematic clarification of the logical structure of such equalizations is directly relevant for solving the above-mentioned ethical questions.

Questions of anthropological dimensions are directly associated, since services in the field of medicine/care are currently performed by humans, as stated above. Thus, the introduction of technical systems replaces the human being in some areas (Decker 2000), technical systems are increasingly involved in human actions, and machines will act in the role of humans in an “as-if” mode; accordingly, technical systems can be only *metaphorically* considered to *actually* take certain (cognitive as well as social) roles of human beings (s. Gutmann 2010). This expansion of the ethical consideration that complies with the double meaning of ἔθος and ἤθος (Gethmann and Sander 1999, 121ff.) finally allows to ask for concepts of man which are—normally implicitly—invested in the construction of the respective technology.

This background is necessary to address issues that go beyond a purely syntactical understanding of technical systems and can be phrased in the following way, taking healthcare services as an example:

But the scope of philosophical consideration extends the limits of ethical and anthropological dimensions by far: Methodological questions become urgent, which are well known from the critical evaluation of AI since the early 60th of the last century (for an extended outline s. Boden 2006). These questions are connected with semantic as well as pragmatic aspects of the “understanding-” and “knowledge-sharing-” potentials of artificial systems: How can a successful care service be classified as “keeping the meaning”? Such a classification does not only require “technical specifications” but also a comprehensive description of the service provided—also considering, for example, friendliness, helpfulness, support etc.

How can this “successful service” be determined as being factually successful? Does this require a long dialogue between “receiver” and “provider” in the sense of a human–machine, machine–human, or a parallel communication via human–human dialogues?

A comprehensive systematic clarification—which is unfortunately only rudimentarily carried out in normal ELSI studies—of the ethical problems of the use (or the prevention of the use) of robotic systems is necessary and should be done under consideration of all three aspects.

This multidisciplinary approach can still be extended. Socio-scientific aspect can be included (Böhle and Pfadenhauer 2011), for example, with empirical studies, to systematically analyse the concrete acceptance on the part of those who provide the service and those who receive the service. This could especially take place on the level of so-called subdisciplines; their relevance for the subject is quite justifiable (Decker and Grunwald 2001).

## Outlook

The multidisciplinary questions described here are studied in the framework of a joint technology assessment of the authors. The perspectives of the different scientific points of view shall be put into an interdisciplinary context with an argumentation aiming at precise recommendations for societal/political decision-making. The development of the interdisciplinary perspective into an “interdisciplinary-disciplinary” perspective is supported by the instrument of “seed texts,” a metaphor that refers to the development of disciplinary texts influenced by interdisciplinary discussions. Under the aspect of quality assurance, it is important to preserve the disciplinary compatibility. At the same time, the seed texts are modified regarding the argumentative support of the resulting recommendations: Therefore, these recommendations are based in a comprehensible way on interdisciplinarily developed lines of arguments. The results of this study might be expected at January 2013.

## Footnotes

1 According to the Verband Deutscher Maschinen- und Anlagenbau e.V. (German Engineering Federation), in 2006 already 50% of all industrial robots had been installed in other sectors than the automotive industry.

2 IFR: http://www.ifr.org/service-robots/ last accessed 30.05.2011.

3 Ichbiah (2005, p. 9ff. and esp. 26ff).

4 Quoted after Christaller et al. (2001, p. 18).

5 Christaller et al. (2001, p. 19).

6 The European Union law also gives a short definition of services in the context of “freedom to provide services” in the treaties of the European Union: Services shall be considered “services” within the meaning of the Treaties where they are provided for remuneration, in so far as they are not governed by the provisions relating to freedom of movement for goods or capital, like activities of commercial character or craftsmen. ‘Services’ shall in particular include: ‘Services’ shall in particular include: (a) activities of an industrial character; (b) activities of a commercial character; (c) activities of craftsmen; (d) activities of the professions., Art. 57 Treaty of the Functioning of the European Union (former Art. 50 of the Treaty of the European Community).

7 Ruckelshausen, A./AMAZONE: BoniRob-… at: www.info.amazone.de/DisplayInfo.aspx?id=13763. As at 30.05.2011.

8 Chocron et al. (2007).

9 ADAC: Statistics of the German automobile club on traffic accidents http://www1.adac.de/Verkehr/Statistiken/default.asp?id=430%26location=2_Verkehr. As at April 2010.

10 BOSCH://www.bosch-kraftfahrzeugtechnik.de/media/de/pdf/fahrsicherheitssysteme_2/vorausschauendesnotbremssystem_hilftauffahrunfllezuvermeidenmindertunfallfolgen.pdf. As at April 2010.

11 NRW: Mobilität in Nordrhein-Westfalen Daten und Fakten 2009.

12 Weltonline: Bundesbürger stehen 535.000 Jahre im Stau, 13.09.2009.

13 Landtag BaWü (2007).

14 http://www.robocuprescue.org/. Last visited 13. September 2011.

15 http://www.robocuprescue.org/ (last accessed on 30.5.2011).

16 http://www.fischertechnik.de/desktopdefault.aspx/tabid-1/61_read-5/usetemplate-2_column_no_pano/ und http://www.technik-lpe.eu/produkte/lego-education/lego-mindstorms.html both 30.5.2011.

17 http://www.firstlegoleague.org/.

18 http://www.iais.fraunhofer.de/uploads/media/Roberta_Mappe.pdf.

19 http://www.nec.co.jp/products/robot/en/index.html. As at 30.5.2011.

20 This idea is not further explained on the website of the manufacturer. However, numerous blog entries and on-line magazines discuss PaPeRo and other robots of its kind, like e.g. Rogun by KornTech, as the new generation of babysitters.

21 http://www.sfb588.uni-karlsruhe.de/about.

22 http://www.ai.rug.nl/robocupathome/ on 30.05.2011.

23 For example: (Weber 2006; Decker 2008).

24 Press release, http://www.xpero.org/portal/readarticle.php?article_id=11 accessed on 30.5.2011.

25 http://www.bmwi.de/BMWi/Navigation/Wirtschaft/dienstleistungswirtschaft,did=239886.html alternativ: http://www.destatis.de, http://www.vgrdl.de.
